# Associations between Gut Microbiota Dysbiosis and Other Risk Factors in Women with a History of Urinary Tract Infections

**DOI:** 10.3390/nu16111753

**Published:** 2024-06-03

**Authors:** Florina Ruța, Mirela Pribac, Elena Mardale, Sara Suciu, Raluca Maior, Simona Bogdan, Călin Avram

**Affiliations:** 1 George Emil Palade University of Medicine Pharmacy, Science and Technology of Targu Mures, 540142 Targu Mures, Romania; florina.ruta@umfst.ro (F.R.); suciu.sara-maria.21@stud.umfst.ro (S.S.); 2 Doctoral School of Biomedical Sciences, University of Oradea, 410087 Oradea, Romania; 3 Regina Maria-Centru Civic, 500036 Brașov, Romania; elena.mardale@reginamaria.ro; 4 Anti-Aging Nutrition Clinic, 540142 Targu Mures, Romania; raluca@clinicanutricare.ro; 5 Amethyst Clinic, 300223 Timisoara, Romania; simona.bogdan@amethyst-radiotherapy.com

**Keywords:** dysbiosis, urinary tract infections, risk factors

## Abstract

(1) Background: Urinary tract infections (UTIs) are among otherwise healthy women represent a problem that requires additional understanding and approaches. Evidencing the link between dysbiosis and UTIs and the associated potential risk factors could lead to therapeutic approaches with increased efficiency under the conditions of reducing the risks associated with antibiotic treatments. The purpose of this study was to evaluate dysbiosis and other potential risk factors in women with a history of urinary tract infections; (2) Methods: Fecal dysbiosis tests were performed comparatively in two groups of women. The first group in-cluded women with recurrent urinary tract infections (rUTI) who had either two or more symp-tomatic episodes of UTI in the previous six months. The second group included women with spo-radic UTIs who did not have >1 UTI during a 12-month period and who did not have another UTI in the last 12 months; (3) Results: An association was shown between intestinal dysbiosis and recurrences of urinary tract infections. Increased body weight was associated with intestinal dysbiosis. Also, the lack of knowledge regarding the risk of using antibiotics and the benefits of probiotics was associated with both dysbiosis and recurrences of urinary tract infections; (4) Conclusions: Dysbiosis can have an impact on the recurrence of urinary tract infections. The risk factors for rUTI and dysbiosis in the sphere of lifestyle are potentially controllable, broadening the perspective for new approaches and changing the paradigm in the treatment of urinary tract infections.

## 1. Introduction

Urinary tract infections (UTIs) are among the most frequent bacterial diseases, affecting an astounding 150 million individuals worldwide each year [1]. Adult women have the highest incidence, at 50–60% [2]. The need for antibiotic treatments for UTIs, which are frequently prescribed and at short intervals, adds significantly to the strain on the health system as well as to the rise in antibiotic resistance [3,4]. Different pathogenic agents are involved in UTIs, and the ascent of bacteria from the intestinal level mostly explains the pathogenesis of the illness. Antibiotic treatment only significantly reduces the count of beneficial bacteria in the gut, which will ultimately lead to an unchecked growth of more dangerous pathogenic strains. This imbalance in the gut microbiota will be the cause of future UTI recurrences due to this imbalance. [5].

The intricate linkages in the microbiota–gut–brain axis [6,7,8,9,10] are comparable to the bidirectional relationship that exists between the kidney and the gut. An increasing body of evidence indicates the involvement of intestinal dysbiosis in the pathophysiology of numerous kidney illnesses, as well as urinary tract infections, and the influence of the intestinal microbiota in the intestine–kidney axis [11].

Uropathogenic bacteria are the primary cause of UTIs. They first infect the periurethral region through uropathogens residing in the intestine, then they colonize the urethra and spread upward to the bladder [12]. More than 80% of UTIs are caused by uropathogenic *Escherichia coli* (UPEC), while other bacteria, including *Klebsiella* spp., *Staphylococcus* spp., *Enterobacter* spp., *Proteus* spp., and *Enterococcus* spp., also play a part [12,13]. Patients with UTIs typically have high concentrations of UPEC strains in their gut, which is assumed to be their source of recurrent urinary tract infections (rUTI) [14,15]. The extragenetic material that UPEC strains have that can encode genes implicated in bacterial pathogenicity (flagella, adhesins, toxins, surface polysaccharides, and iron acquisition factors) sets them apart from commensal *E. coli* [16,17].

The intestine is a natural habitat for many bacteria, including *Escherichia coli* (*E. coli*). These bacteria can reach the urinary tract in several ways, such as through migration or contamination of the urinary tract through various mechanisms. *E. coli* is one of the main causes of urinary tract infections (UTIs). Based on the assumption that the origin of bacterial infections in the urinary tract is the overgrowth of certain bacteria in the intestinal tract, Magruder et al. demonstrated an increased abundance of *Escherichia coli* in the intestine, the bacteria most frequently involved in UTIs. The authors associated this gut dysbiosis, characterized by the overgrowth of conditionally pathogenic strains, as a risk of urinary infections. Furthermore, strain analysis identified similarities between species found in the intestine and those found in the urinary tract in the investigated subjects, supporting the theory of the gut microbiota–UTI axis. Among the species frequently involved in UTIs are *Klebsiella* spp., *Staphylococcus* spp., and *Streptococcus* spp., but with less significant evidence regarding the association between their abundant intestinal levels and their involvement in UTIs [18,19].

Nutritional therapies combined with probiotic and plant-based supplementation at the gastrointestinal level can significantly enhance intestinal health, according to Dr. Leo Galland (Director of the Foundation for Integrative Medicine, New York, NY, USA) [20]. In the context of actions that combine the effect of supplements with antibacterial and probiotic action with dietary measures, the gut can dramatically improve its condition through its ability to regenerate and restore intestinal cells [21,22,23]. This allows the intestine to control the proliferation of UPEC and other conditionally pathogenic bacteria.

In this study, a group of young, otherwise healthy women were asked to assess the relationship between intestinal dysbiosis and recurrent urinary tract infections (UTIs) as well as other characteristics that promote UTI recurrences.

The purpose of this study was to evaluate dysbiosis and other potential risk factors in women with a history of repeated urinary tract infections.

## 2. Materials and Methods

### 2.1. Selection of Study Groups

The computerized databases of family medicine and ambulatory practices in Romania were utilized to choose study participants. Women aged 18–45 who have been diagnosed with a UTI in the past five years were selected. Inclusion in this study was on a volunteer basis. Women aged 18–45 with a diagnosis of UTI, registered in the last five years, were selected. Women who accepted voluntary participation were part of this study. Further selection was made choosing the first study group of women with recurrent urinary tract infections (rUTIs), defined as those who had either two or more symptomatic UTI episodes in the previous six months or more than three UTI episodes in the previous year. Based on urine culture, ≤103 cfu/mL of uropathogenic bacteria was the definition of a UTI. Urine cultures for at least one UTI were required for the final selection of patients. The second study group consisted of women who did not have >1 UTI during a 12-month period and who did not have another UTI in the last 12 months and women with a history of sporadic UTI (nonrecurrent urinary tract infection nUTI) in the last five years. 

Exclusion criteria included women who did not voluntarily agree to participate, women out of the proposed age range, women who were pregnant or nursing, and women with comorbidities that could influence in any way the occurance of UTIs such as renal lithiasis (kidney stones), neurological conditions, immunosuppressive states, surgical interventions of the urinary tract, diabetes, and constipation. We did not include women outside the age range chosen by us, those who refused to participate, and those with diseases that can increase the risk of urinary tract infections (kidney stones, neurological diseases, immunosuppressed states, surgical interventions of the urinary tract, diabetes, constipation). Likewise, women who were pregnant or breastfeeding at the time of ICU registration were not included.

### 2.2. Laboratory Determinations 

This study was carried out in 2023. All patients underwent a fecal dysbiosis test at a single specialized laboratory. Dysbiosis analysis consisted of fecal culture. For harvesting, a single harvester was used; the harvested amount of biological product was 1 g; the harvesting was done at least 7 days after the end of an antibiotic treatment. All stool samples were delivered to the laboratory on the day of collection within a predetermined time frame, and the processing of the samples was carried out at equal time intervals for all subjects. All fecal samples were delivered to the laboratory on the day of collection, within a predetermined time interval, and the sample processing was done at an equal time interval for all subjects. We considered dysbiosis present at a Flora Index level ≥ 6.

### 2.3. Examination of Lifestyle, Diet, and History of UTIs

In addition, the study participants filled out a questionnaire to evaluate their nutritional health, eating habits, history of antibiotic resistance after receiving repeated antibiotic treatments, and lifestyle risk factors such drinking alcohol, smoking, and coffee consumption. Less than one liter of water per day, mostly carbonated mineral water consumption, daily consumption of sweet juices with reference to sweet soft drinks, or more than 250 mL of freshly squeezed fruit juice were used to monitor water intake and food consumption behavior considered at risk. To quantify the consumption of vegetables, excluding potatoes, benchmarks were offered such as 1 serving = 1/2 cup of greens or 1 cup of green leaves, while for fruit 1 serving = 1 medium-sized fruit or 120 mL of freshly squeezed juice 100%. For whole grains 1 serving = 1 slice of 100% whole grain bread, 1 cup of whole grains, high fiber grains, oatmeal, 3–4 whole meal saltine crackers, ½ cup wild rice or whole meal pasta. For the consumption of salty foods, reference was made to pickles/soups in envelopes/ready-made/salty seeds), and for hypercaloric foods, there was the possibility of selection for cookies, cake, biscuits, wafers, pastry products, doughnuts, muffins, chocolate, candy, ice cream. Questions related to the daily consumption of meat and processed meat derivatives as well as dairy products were added to the questionnaire. Based on the reported data, the body mass index was calculated.

### 2.4. Statistical Data Analysis

The database was created in Microsoft Excel 2010, and the statistical analysis was performed in the statistical program GraphPad Prism version 10. For the dichotomous variables, we identified the number and the percentage, and for the numerical variables, we calculated the mean and the standard deviation. The numerical data were checked for normality using the Shapiro–Wilk test [24] and depending on this result we applied parametric or non-parametric tests. Two multivariable logistic regression models were used to assess the odds of having dysbiosis versus no dysbiosis and the odds of having recurrent UTIs versus not having UTIs. The significance threshold was set at 0.05.

## 3. Results

There were 753 participants in this study. The group’s average age was 37.65 years, and the majority of responders (73%) had normal nutritional status (BMI = 18–24.9 m^2^/kg) (Table 1).

In 537 (71%) of the women, gut dysbiosis was found. We identified several factors associated with dysbiosis, including the use of other drinks (carbonated water: *p* = 0.0033, juices: *p* < 0.0001, energy drinks: *p* = 0.0001), multiresistance to antibiotics as a result of repeated antibiotic treatments (*p* < 0.0001), and recurrences of UTI prior to the last year (*p* < 0.0001).

Of those who reported consuming salty foods, over half had dysbiosis (60%). Around half reported consuming fewer fruits, vegetables, and cereals (54%), as well as 40% reporting lower consumption of all three. Additionally, a statistically significant correlation (*p* = 0.0001) was found between the consumption of hypercaloric diets high in added sugars and saturated fats. Alcohol use (*p* < 0.0001) and meat eating (*p* = 0.0004) were found to have an impact on dysbiosis. (Table 2).

Dysbiosis was influenced by the use of dietary supplements to avoid UTIs (<0.0001). Data on the use of antibiotics, as well as the function, mode, and length of probiotic administration, were statistically significant (*p* < 0.0001) in their associations with dysbiosis (Table 2).

When dysbiosis was present, recurrent urinary tract infections and drug resistance were more common. Moreover, dysbiosis has been linked to decreased water intake. The existence of dysbiosis was statistically substantially correlated with the lack of knowledge on the impact of food supplements advised in UTI cases and the impact of antibiotics on the beneficial bacterial microflora in the body (Table 3).

Of the women who were chosen in total, 42% had an rUTI, and 76% had dysbiosis. Antibiotic resistance (*p* < 0.0001) and dysbiosis were also linked to rUTIs (*p* < 0.0001).

The majority of women who abstain from carbonated and stimulating drinks seem to have less rUTIs. Consuming high-calorie foods with added sugar and fat was linked to UTIs; the same association was found while eating food supplements that had a prophylactic effect on UTIs (Table 4).

Table 5 lists the risk factors for rUTIs, including the existence of dysbiosis, multiple antibiotic resistance, a lack of usage of supplements that can prevent rUTIs, and ignorance about the best way and length of time to supplement with probiotics.

## 4. Discussion

The risk of intestinal mucosal hyperpermeability is increased by modern lifestyle habits, which is typified by an excessively high intake of simple carbohydrates and fats, low fiber, and a high concentration of pesticides and insecticides in food, chronic alcohol consumption, heavy metals in food and water, and sometimes unnecessary antibiotic treatments. An imbalance of bacteria in the gut is known as intestinal dysbiosis. Intestinal microorganisms, commonly referred to as intestinal flora, are mostly composed of different kinds of bacteria and, to a lesser degree, fungi, and protozoa. The body’s immunity and digestion depend on the bacteria living in the gastrointestinal tract. A lack of diversity or an imbalance in the gut’s microbial population might be characterized as gut dysbiosis. Accordingly, illnesses related to the digestive system and other systemic symptoms arise from gut dysbiosis [25]. The findings of our investigation support the generally held belief that the gut microbiota plays a role in the onset and progression of obesity. The changes in diet and lifestyle trigger a fast response from the gut bacteria.

Even while the standard therapy for UTIs involves the use of antibiotics, the side effects are still characterized by the long-term disruption of the normal intestinal microbiota and the subsequent rise of multi-resistant microorganisms. For both preventive and therapeutic purposes, this condition necessitates the identification of alternate and complementary therapeutic choices in the ICU approach [26]. Consequently, it has been established that one of the primary causes of UTIs is gut dysbiosis [27]. Our study highlighted in the selected group, a majority of women with intestinal dysbiosis (over 70%), and the prevalence of dysbiosis among women with rUTIs was increased (over 70%). Multi-resistance to antibiotics, as a result of repeated treatments, confirmed by testing the sensitivity of germs to antibiotics, was statistically significantly associated with both dysbiosis and rUTIs. A percentage of 39% of patients with dysbiosis declared a more distant history of frequent urinary infections, with a statistically significant association between UTI relapses and the presence of dysbiosis.

Recent data have shown that the human intestinal microbiota is sensitive to food preservatives [28] which can cause excessive growth of proteobacteria [29]. Food emulsifiers can also alter the composition of the human intestinal microbiota with the initiation of intestinal inflammation [30]. Artificial, noncaloric sweeteners that have been recommended as sugar substitutes for the purpose of preventing metabolic syndrome have been identified to induce dysbiosis and glucose intolerance in a microbiota-dependent manner, actually generating negative metabolic effects instead of the intended protector [31]. Sweet and energizing drinks have the potential to influence the status of the intestinal flora, through the content of such substances, as is evident from the data of our study. The consumption of less than one liter of water per day was associated with dysbiosis (*p* = 0.0031), the effect being due to either an insufficient liquid intake or supplementing the intake of liquids with drinks other than water.

Only 12% of the structural variation in gut microbiota may be attributed to genetic changes; 57% may be explained by dietary changes [32]. This demonstrates how nutrition plays a major influence in regulating gut flora. Dysbiosis is brought on by the hypercaloric Western diet, which is high in sugar and fat [33]. This was also shown in our study by the propensity for alcohol and processed meat to produce dysbiosis. We modified the Rapid Eating Assessment for Patients questionnaire to include items about quantity and frequency of food consumption at risk.

Drinking alcohol as a risk factor was defined as consuming more than one serving (150 mL) per day, processed meat as more than 100 g per day, whole grains and vegetables as less than three servings per day, and fruit as fewer than two servings per day [34].

Alterations in the intake of macronutrients can lead to rapid and profound alterations in the gut flora. These modifications have important physiological ramifications. For instance, diets high in simple carbohydrates can damage the intestinal barrier, induce inflammation, and have a negative impact on host metabolism. It has been shown that the habits of our participant of consuming processed meals that are high in calories, added sugar, fat, or salt has the potential to affect dysbiosis. Excessive fruit consumption, salt intake, and high-calorie meal consumption all produced similar outcomes. Dysbiosis and multi-resistance to antibiotics (*p* = 0.0005) are factors that predict rUTI (*p* = 0.0001). Dietary habits that may have an impact on rUTI include consuming energy drinks and carbonated water, as well as consuming insufficient amounts of fruits, vegetables, whole grains, and processed foods high in calories.

A useful tactic in the management and prevention of illnesses, including UTIs, may involve altering the gut microbiota. Restoring the gut microbiota’s balance and lowering pathogen abundance are two benefits of using dietary supplements that contain commensal bacteria as probiotics [35].

In the context of a randomized, double-blind, placebo-controlled pilot study, Koradia et al. reported the effects of administering a commercially available probiotic product made of two strains of *Lactobacillus* and cranberry extract, which significantly reduced the number of recurrent urinary tract infections in young premenopausal women when compared to a placebo product [36]. The delivery of lactobacilli did not promote bacterial resistance to antibiotics, which was a major benefit of using probiotics as opposed to antibiotic treatment [37].

Probiotics, especially those with strains from the Lactobacillaceae family, may be useful in preventing urinary tract infections, as *Lactobacillus* spp. prevent the adherence, growth, and colonization of uropathogenic bacteria. Studies show that healthy microbial populations of *Lactobacillus* spp. have a strong inhibitory effect on *E. coli* [38]. Also, in some cases, research has shown that certain strains of probiotics, such as *L. salivarius* with enteric release, reach the level of the urinary and vaginal microbiota, with a direct effect on the respective devices [39]. On the other hand, bifidobacteria improve the use of lactose (in the context of the genetic and physiological deficiency of lactase deficiency) and also help to stabilize the intestinal mucosal barrier [40]. Prebiotics promote the growth of lactic acid bacteria and the production of butyrate and lactate, the main colonic metabolites with a role in the balance of the microbiome. Probiotics, prebiotics are therapeutic means associated with the modification of the intestinal microbiota [41]. A risk factor for both dysbiosis (*p* = 0.0182) and rUTI (*p* = 0.0153) was knowledge of the potential use of dietary supplements with a preventative role of UTI (*p* < 0.001). Another risk factor for dysbiosis was the lack of knowledge about how antibiotics affect the beneficial bacteria in the body (*p* = 0.0049).

The synergistic effect of the essential oils (EO) of Oregano, Melaleuca, Lemon, Lemongrass, Peppermint, and Thyme has been studied for its antibacterial, antifungal, and gut-modulating effects. These EOs have broad-spectrum antibacterial and antifungal potential and can support the achievement of an unfavorable environment for the conditional proliferation of pathogens that can disrupt homeostasis in the gastrointestinal tract [42]. Moreover, probiotic nutritional support formulas containing active strains of *Bifidobacterium lactis*, *B. bifidum*, *B. longum*, and *Lactobacillus acidophilus*, *L. salivarious*, and *L. casei* have been shown to contribute to the growth of beneficial bacterial colonies, which in turn enhance local immunity, promote the synthesis of vitamins B1, B2, PP, and K, and enhance their metabolism [43].

Given the significance of preserving a balanced microbiota and a healthy gut on urinary tract health, the nutritional therapy approach—which entails assessing its effects—may be given consideration. Patients and primary care physicians need to be aware of the necessity of reevaluating the long-term management of UTI recurrences and incorporating an interdisciplinary approach through measures that promote the balance of the intestinal microbiome.

Approximately 1000 different kinds of bacteria, fungus, and other microbes can be found in the intestine [44].

An adult’s microbiota is distinct and has remained mostly constant over time. A balanced microbiota is characterized by a preponderance of obligately anaerobic bacteria belonging to the taxonomic subdivisions Firmicutes (Clostridia, Ruminococcaceae, Eubacteriaceae) and Bacteroidetes (Bacteroidaceae, Prevotellaceae). Enterobacteriaceae and *Fusobacteria* spp. are examples of actinobacteria and proteobacteria. To have a healthy intestinal flora, one must have a very diverse microbiome. Dysbiosis, which manifests as an increase in potentially harmful bacterial species (those not in the Bacteroidia or Clostridia classes), is linked to a decline in physiological diversity [45].

**Figure 1:**
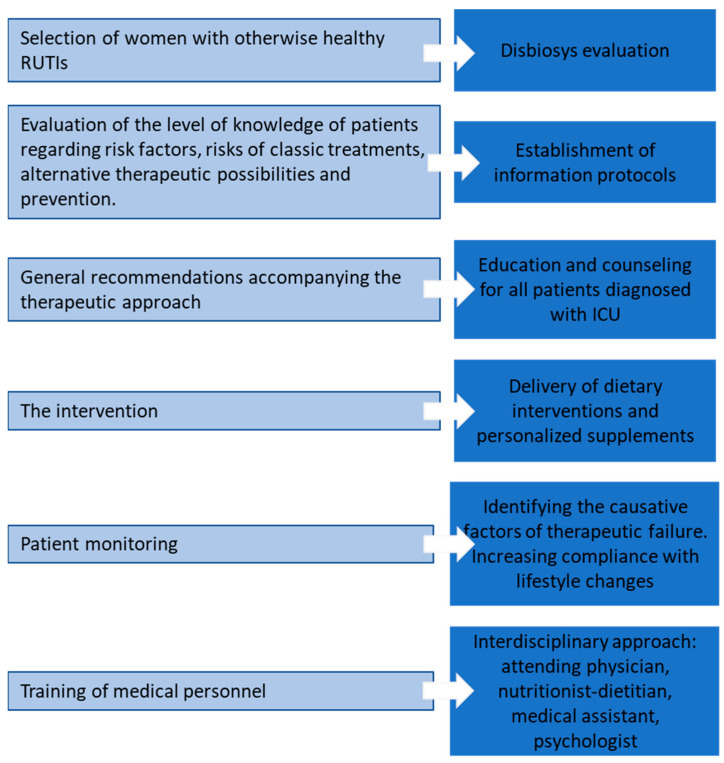
Topics and objectives of the rUTI management program.

Correcting these risk factors through diet changes, the use of food supplements [46], lifestyle improvements, training medical personnel to identify these risk factors and inform patients and educate them for healthy behavior [47,48] can represent the preamble of alternative strategies for the prevention and treatment of recurrences of urinary infections, in people in good health, in order to limit the use of antibiotics (Figure 1).

The results of allopathic therapeutic techniques are not very encouraging, especially when it comes to recurrences, despite the studies that have been conducted on UPEC strains in an attempt to comprehend the mechanisms of bacterial migration, adaption, and multiplication. The risk of recurrence remains relatively high even with an accurate microbiological diagnosis and prompt use of traditional treatment, thereby underscoring the significance of intestinal microbiota health.

We believe that antibiotic therapy for UTIs only dramatically increases the risk of reducing beneficial bacteria from the gut, which actually contributes to the uncontrolled proliferation of more dangerous pathogenic strains (including *E. coli*), which supports future recurrences. Due to the importance of maintaining a healthy gut and a balanced microbiome, the therapeutic approach of UTI could be oriented, at least concurrently with the antibiogram, towards microbiome testing and management. Both patients and general practitioners must understand the need to reconsider the management of UTI recurrences, to adapt [49,50,51,52] to new long-term therapeutic perspectives and the inclusion of an integrative approach to this problem through interventions aimed at supporting the microbiome, both for otherwise healthy individuals and for vulnerable ones, in which antibiotic treatment also involves other potential risks, such as the case of women during pregnancy [53]. Microbiota homeostasis and the treatment of obesity and related comorbidities may benefit from gut microbiome manipulation through dietary modifications, lifestyle adjustments, prebiotics, and probiotics [54].

Urologists rarely consider the role of microbes beyond their involvement in infectious pathology of the urinary tract specifically. In the future, all medical practitioners involved in treating urinary tract infections will need to consider the potential influences of the gut microbiota in both diagnosis and treatment. New research in the field may provide opportunities to anticipate the risk of developing urological diseases and enable the development of innovative therapeutic strategies with interdisciplinary involvement.

The limitations of this study consisted of the high cost of the fecal dysbiosis test and, in some cases, given the large number of participants, the challenges concerning collecting and delivering the biological samples to the laboratory. Another limitation would be the possible subjectivity of the participant when completing the questionnaire, answers being subject to personal interpretation of symptoms or other aspects that have multiple variables.

## 5. Conclusions

Dysbiosis can have an impact on the recurrence of urinary tract infections. Risk factors for rUTI and gut dysbiosis are both potentially controllable. These risk factors either directly affect both conditions or only one of them, gut dysbiosis, for example, which then maintains rUTI on the long term, supporting a never-ending vicious circle.

Since the gastrointestinal tract is one of the main anatomical regions implicated in the pathophysiology of UTIs, novel strategies and a paradigm shift in UTI treatment are necessary.

## Figures and Tables

**Table 1:** General characteristics of the batch.

N = 753	Dysbiosis Yes (N = 537)	Dysbiosis No (N = 216)	*p* Value	rUTI (N = 317)	nUTI (N = 436)	*p* Value
Age Mean (SD)	37.65 (8.72)	35.83 (8.53)	0.1952 *	38.27 (8.51)	36.30 (8.75)	0.1234 **
Weight Mean (SD)	64.36 (11.91)	65.39 (12.34)	0.6699 **	63.48 (11.19)	65.51 (12.56)	0.2248 **
Height Mean (SD)	165.23 (6.11)	166.93 (6.71)	0.0963 *	165.4 (5.89)	165.9 (6.63)	0.5646 *
BMI > 25 m^2^/Kg BMI = 18–24.9 m^2^/kg	185 (34%) 352 (66%)	50 (23%) 166 (77%)	0.002 ***	77 (24%) 240 (76%)	128 (29%) 308 (71%)	0.1230 ***

* Unpaired *t* test; ** Mann Whitney test; *** chi square test.

**Table 2:** Distribution according to dysbiosis and potential risk factors.

	Dysbiosis Yes (N = 537)	Dysbiosis No (N = 216)	*p* Value
Recurrences of UTI before the last year • Yes • No	297 (55%) 240 (45%)	85 (39%) 131 (61%)	<0.0001
The presence of multi-resistance to antibiotics as a result of antibiotic treatments, confirmed by antibiogram • Yes • No	113 (21%) 424 (79%)	12 (6%) 204 (94%)	<0.0001
Consumption of less than 1 L of water per day • Yes • No	236 (44%) 301 (56%)	144 (67%) 72 (33%)	<0.0001
Daily consumption of sweet juices • Yes • No	137 (26%) 400 (74%)	88 (41%) 128 (59%)	<0.0001
Daily consumption of energy drinks • Yes • No	61 (11%) 476 (89%)	48 (22%) 168 (78%)	0.0001
Less than 3 servings of vegetables consumed daily • Yes • No	285 (53%) 252 (47%)	124 (57%) 92 (43%)	0.2801
Less than 2 servings of fruit consumed daily • Yes • No	235 (44%) 302 (56%)	126 (58%) 90 (42%)	0.0003
Less than 3 servings of whole grains consumed daily • Yes • No	233 (43%) 304 (57%)	92 (43%) 124 (57%)	0.8418
Habitual consumption of salty foods • Yes • No	113 (21%) 424 (79%)	76 (35%) 140 (65%)	<0.0001
Eating high-calorie, processed foods with added sugar and fat • Yes • No	281 (52%) 256 (48%)	148 (69%) 68 (31%)	<0.0001
Daily consumption of meat and processed meat derivatives • Yes • No	353 (66%) 184 (34%)	112 (52%) 104 (48%)	0.0004
Daily dairy consumption • Yes • No	293 (55%) 244 (45%)	104 (48%) 112 (52%)	0.1108
The use of dietary supplements for preventive purposes, during the period without UTI • Yes • No	148 (28%) 389 (72%)	92 (43%) 124 (57%)	<0.0001
Information received about the effect of antibiotics on the good bacteria in the body (risk of dysbiosis) • Yes • No	312 (58%) 225 (42%)	160 (74%) 56 (26%)	<0.0001
Compliance with the antibiotic administration schedule and doses • Yes • No	485 (90%) 52 (10%)	184 (85%) 32 (15%)	0.0431
Information received about food supplements recommended in ITU • Yes • No	324 (60%) 213 (40%)	96 (44%) 120 (56%)	<0.0001
Information received about the role of probiotics in the treatment of UTI • Yes • No	424 (79%) 113 (21%)	140 (65%) 76 (35%)	<0.0001
Information received about the method and duration of administration of probiotics. • Yes • No	440 (82%) 97 (18%)	148 (69%) 68 (31%)	<0.0001
Compliance with the recommendations for the administration of probiotics • Yes • No	433 (81%) 104 (19%)	164 (76%) 52 (24%)	0.1494
Drinking coffee in the morning on an empty stomach? • Yes • No	289 (54%) 248 (46%)	108 (50%) 108 (50%)	0.3426
Smoker • Yes • No	133 (25%) 404 (75%)	68 (31%) 148 (69%)	0.0596
Alcohol consumption • Yes • No	64 (12%) 473 (88%)	54 (25%) 162 (75%)	<0.0001

**Table 3:** Risk factors associated with gut dysbiosis.

	ORR	95% CI	*p* Value
Recurrences of UTI	1.8447	0.9205–3.6967	0.0443
The presence of multi-resistance to antibiotics as a result of antibiotic treatments, confirmed by antibiogram	3.7182	1.0325–13.3903	0.0445
Consumption of less than 1 L of water per day	0.3467	0.1718–0.6994	0.0031
Predominant consumption of carbonated mineral water	0.7179	0.2942–1.7517	0.4664
Daily consumption of sweet juices	0.7874	0.3030–2.0463	0.6238
Daily consumption of energy drinks	0.4318	0.1396–1.3358	0.1450
Eating high-calorie, processed foods with added sugar and fat	0.6706	0.3006–1.4961	0.3291
Daily consumption of meat and processed derivatives	1.6383	0.7148–3.7551	0.2434
Daily dairy consumption	1.0407	0.4583–2.3630	0.9241
Lack of use of dietary supplements for preventive purposes, during the period without UTI	2.1268	0.9456–4.7837	0.0681
Lack of information about the effect of antibiotics on the good bacteria in the body (risk of dysbiosis)	3.9805	1.5202–10.4224	0.0049
Lack of information about food supplements recommended in ITU	0.3519	0.1480–0.8371	0.0182
Lack of information about the role of probiotics in the treatment of UTI	0.5191	0.2461–1.0949	0.0851
Lack of information about the method and duration of administration of probiotics	0.3749	0.1286–1.0935	0.0724

**Table 4:** Distribution according to dysbiosis and potential risk factors.

	rUTIs (N = 317)	nUTI (N = 436)	*p* Value
Dysbiosis present • Yes • No	241 (76%) 76 (24%)	272 (62%) 164 (38%)	<0.0001
The presence of multi-resistance to antibiotics as a result of antibiotic treatments, confirmed by antibiogram • Yes • No	93 (29%) 224 (71%)	32 (7%) 404 (93%)	<0.0001
Consumption of less than 1 L of water per day • Yes • No	157 (50%) 160 (50%)	224 (51%) 212 (49%)	<0.0001
Predominant consumption of carbonated mineral water • Yes • No	122 (38%) 195 (62%)	88 (20%) 348 (80%)	<0.0001
Daily consumption of sweet juices • Yes • No	101 (32%) 216 (68%)	136 (31%) 300 (69%)	0.8454
Daily consumption of energy drinks • Yes • No	69 (22%) 248 (78%)	40 (9%) 396 (91%)	0.0001
Less than 3 servings of vegetables consumed daily • Yes • No	149 (47%) 168 (53%)	256 (59%) 180 (41%)	0.0015
Less than 2 servings of fruit consumed daily • Yes • No	139 (44%) 178 (56%)	256 (59%) 180 (41%)	<0.0001
Less than 3 servings of whole grains consumed daily • Yes • No	117 (37%) 200 (63%)	212 (49%) 224 (51%)	0.0014
Habitual consumption of salty foods • Yes • No	80 (25%) 237 (75%)	108 (25%) 328 (75%)	0.8840
Eating high-calorie, processed foods with added sugar and fat • Yes • No	201 (63%) 116 (37%)	98 (22%) 338 (78%)	<0.0001
Daily consumption of meat and processed meat derivatives • Yes • No	267 (84%) 50 (16%)	195 (45%) 241 (55%)	<0.0001
Daily dairy consumption • Yes • No	207 (65%) 110 (35%)	109 (25%) 327 (75%)	<0.0001
The use of dietary supplements for preventive purposes, during the period without UTI • Yes • No	106 (33%) 211 (67%)	84 (19%) 352 (81%)	<0.0001
Information received about the effect of antibiotics on the good bacteria in the body (risk of dysbiosis) • Yes • No	204 (64%) 113 (36%)	269 (62%) 167 (38%)	0.4565
Compliance with the antibiotic administration schedule and doses • Yes • No	268 (85%) 49 (15%)	400 (92%) 36 (8%)	0.0021
Information received about food supplements recommended in ITU • Yes • No	196 (62%) 121 (38%)	224 (51%) 212 (49%)	0.0044
Information received about the role of probiotics in the treatment of UTI • Yes • No	257 (81%) 60 (19%)	308 (71%) 128 (29%)	0.0011
Information received about the method and duration of administration of probiotics. • Yes • No	276 (87%) 41 (13%)	312 (72%) 124 (28%)	<0.0001
Compliance with the recommendations for the administration of probiotics • Yes • No	237 (75%) 80 (25%)	360 (83%) 76 (17%)	0.0091
Drinking coffee in the morning on an empty stomach? • Yes • No	145 (46%) 172 (54%)	248 (57%) 188 (43%)	0.0025
Smoker • Yes • No	93 (29%) 224 (71%)	108 (25%) 329 (75%)	0.1564
Alcohol consumption • Yes • No	24 (8%) 293 (92%)	68 (16%) 368 (84%)	0.0009

**Table 5:** Risk factors associated with urinary infection.

	ORR	95% CI	*p* Value
Dysbiosis present	5.9659	2.4077–14.7821	0.0001
The presence of multi-resistance to antibiotics as a result of antibiotic treatments, confirmed by antibiogram	5.3147	2.0726–13.6280	0.0005
Consumption of less than 1 L of water per day	1.1504	0.6240–2.1211	0.6534
Predominant consumption of carbonated mineral water	1.6897	0.7568–3.7727	0.2006
Daily consumption of sweet juices	0.9420	0.4301–2.0633	0.8813
Daily consumption of energy drinks	2.3870	0.7958–7.1602	0.1206
Less than 3 servings of vegetables consumed daily	1.3653	0.6624–2.8142	0.3987
Less than 2 servings of fruit consumed daily	0.8035	0.3888–1.6604	0.5546
Less than 3 servings of whole grains consumed daily	0.5509	0.2739–1.1082	0.0945
Eating high-calorie, processed foods with added sugar and fat	1.3893	0.6957–2.7744	0.3515
Daily consumption of meat and processed derivatives	1.4385	0.6118–3.3820	0.4045
Daily dairy consumption	0.4779	0.2060–1.1085	0.0854
Lack of use of dietary supplements for preventive purposes, during the period without UTI	0.1145	0.0501–0.2617	<0.0001
Lack of information about food supplements recommended in ITU	1.3259	0.6096–2.8836	0.4767
Lack of information about the role of probiotics in the treatment of UTI	1.3070	0.4305–3.9680	0.6366
Lack of information about the method and duration of administration of probiotics	0.2166	0.0629–0.7455	0.0153

## Data Availability

The original contributions presented in the study are included in the article, further inquiries can be directed to the corresponding author.
